# Cohort Profile: The 100 Million Brazilian Cohort

**DOI:** 10.1093/ije/dyab213

**Published:** 2021-12-18

**Authors:** Mauricio L Barreto, Maria Yury Ichihara, Julia M Pescarini, M Sanni Ali, Gabriela L Borges, Rosemeire L Fiaccone, Rita de Cássia Ribeiro-Silva, Carlos A Teles, Daniela Almeida, Samila Sena, Roberto P Carreiro, Liliana Cabral, Bethania A Almeida, George C G Barbosa, Robespierre Pita, Marcos E Barreto, Andre A F Mendes, Dandara O Ramos, Elizabeth B Brickley, Nivea Bispo, Daiane B Machado, Enny S Paixao, Laura C Rodrigues, Liam Smeeth

**Affiliations:** Centre for Data and Knowledge Integration for Health (CIDACS), Fundação Oswaldo Cruz, Salvador, Brazil; Institute of Collective Health, Federal University of Bahia (UFBA), Salvador, Brazil; Centre for Data and Knowledge Integration for Health (CIDACS), Fundação Oswaldo Cruz, Salvador, Brazil; Institute of Collective Health, Federal University of Bahia (UFBA), Salvador, Brazil; Centre for Data and Knowledge Integration for Health (CIDACS), Fundação Oswaldo Cruz, Salvador, Brazil; Faculty of Epidemiology and Population Health, London School of Hygiene and Tropical Medicine, London, UK; Centre for Data and Knowledge Integration for Health (CIDACS), Fundação Oswaldo Cruz, Salvador, Brazil; Faculty of Epidemiology and Population Health, London School of Hygiene and Tropical Medicine, London, UK; Nuffield Department of Orthopaedics, Rheumatology and Musculoskeletal Sciences, Center for Statistics in Medicine, University of Oxford, Oxford, UK; Centre for Data and Knowledge Integration for Health (CIDACS), Fundação Oswaldo Cruz, Salvador, Brazil; Centre for Data and Knowledge Integration for Health (CIDACS), Fundação Oswaldo Cruz, Salvador, Brazil; Department of Statistics, Federal University of Bahia, Salvador, Brazil; Centre for Data and Knowledge Integration for Health (CIDACS), Fundação Oswaldo Cruz, Salvador, Brazil; Department of Nutrition, Federal University of Bahia, Salvador, Brazil; Centre for Data and Knowledge Integration for Health (CIDACS), Fundação Oswaldo Cruz, Salvador, Brazil; Centre for Data and Knowledge Integration for Health (CIDACS), Fundação Oswaldo Cruz, Salvador, Brazil; Centre for Data and Knowledge Integration for Health (CIDACS), Fundação Oswaldo Cruz, Salvador, Brazil; Centre for Data and Knowledge Integration for Health (CIDACS), Fundação Oswaldo Cruz, Salvador, Brazil; Centre for Data and Knowledge Integration for Health (CIDACS), Fundação Oswaldo Cruz, Salvador, Brazil; Centre for Data and Knowledge Integration for Health (CIDACS), Fundação Oswaldo Cruz, Salvador, Brazil; Centre for Data and Knowledge Integration for Health (CIDACS), Fundação Oswaldo Cruz, Salvador, Brazil; Centre for Data and Knowledge Integration for Health (CIDACS), Fundação Oswaldo Cruz, Salvador, Brazil; Centre for Data and Knowledge Integration for Health (CIDACS), Fundação Oswaldo Cruz, Salvador, Brazil; Department of Statistics, London School of Economics and Political Science, London, UK; Centre for Data and Knowledge Integration for Health (CIDACS), Fundação Oswaldo Cruz, Salvador, Brazil; Centre for Data and Knowledge Integration for Health (CIDACS), Fundação Oswaldo Cruz, Salvador, Brazil; Institute of Collective Health, Federal University of Bahia (UFBA), Salvador, Brazil; Faculty of Epidemiology and Population Health, London School of Hygiene and Tropical Medicine, London, UK; Centre for Data and Knowledge Integration for Health (CIDACS), Fundação Oswaldo Cruz, Salvador, Brazil; Department of Statistics, Federal University of Bahia, Salvador, Brazil; Centre for Data and Knowledge Integration for Health (CIDACS), Fundação Oswaldo Cruz, Salvador, Brazil; Centre for Data and Knowledge Integration for Health (CIDACS), Fundação Oswaldo Cruz, Salvador, Brazil; Faculty of Epidemiology and Population Health, London School of Hygiene and Tropical Medicine, London, UK; Centre for Data and Knowledge Integration for Health (CIDACS), Fundação Oswaldo Cruz, Salvador, Brazil; Faculty of Epidemiology and Population Health, London School of Hygiene and Tropical Medicine, London, UK; Faculty of Epidemiology and Population Health, London School of Hygiene and Tropical Medicine, London, UK

Key Features

- The creation of The 100 Million Brazilian Cohort was motivated by the availability of high quality but dispersed social and health databases in Brazil and the need to integrate data and evaluate the impact of policies aiming to improve the social determinants of health (e.g. social protection policies) on health outcomes, overall and in subgroups of interest in a dynamic cohort.
- The baseline of The 100 Million Brazilian Cohort comprises 131 697 800 low-income individuals in 35 358 415 families from 2011 to 2018. The Cohort population is mostly composed of children and young adults, with a higher proportion of females than the general Brazilian population, who identify themselves as Brown and live in the urban area of the country.
- Exposure to social protection and the follow-up of individuals are obtained through: (i) deterministic linkage using the Social Identification Number (NIS) to link the Cohort baseline to social protection programmes and to periodically renewed socioeconomic information in Cadatro Único datasets; and/or (ii) non-deterministic linkage using the CIDACS-RL non-deterministic linkage tool, to link the Cohort baseline to administrative health care datasets such as mortality (Mortality Information System, SIM), disease notification (Information System for Notifiable Diseases, SINAN), birth information (Live Birth Information System, SINASC) and nutrition status (Food and Nutrition Surveillance System, SISVAN).
- So far, studies have used The 100 Million Brazilian Cohort to investigate the socioeconomic and demographic determinants of leprosy, leprosy treatment outcomes and low birthweight and to evaluate the impact of the Bolsa Familia Programme (BFP) on leprosy and child mortality. Other studies are now being conducted that are of utmost relevance to the health inequalities of Brazil and many low- and middle-income countries, and many research opportunities are being opened up with the linkage of a range of health outcomes.

## Why was the cohort set up?

Large social inequalities and poverty are major historical characteristics of Latin America in general and Brazil in particular. In the past two decades, Brazil has experienced substantial social and health changes, including significant reductions in poverty and inequalities. Although social protection has been one of the major contributors to improved living conditions in this country, the benefits may be facing a reversal with recent political changes in several countries with governments prone to privilege ‘austerity’ over ‘social protection’ policies.^1–3^ Among the programmes implemented to expand social protection, the most prominent one is the Conditional Cash Transfer Programme, the ‘Bolsa Família Programme’ (BFP), that transfers cash directly to poor households. Additional social protection programmes include the Housing Programme(‘Minha Casa Minha Vida’), the Access to Water Programme (‘Cisternas’) and the Light for All Programme (‘Luz Para Todos’) to improve access to electricity in rural communities, among others.

The social determinants of health, the conditions in which people are born, grow, work, live and age, are important causes of health inequities and influence the onset and evolution of many different aspects of illness and health.^4–7^ Whereas there is substantial evidence on the effect of different social and economic factors on health, little is known about the health impact of policies targeted to modify these socioeconomic determinants.^8–10^ Evaluation of the effects of societal, economic and environmental policies and interventions on health outcomes is complex and requires approaches different from the traditional methods such as randomixed control trials (RCTs) and observational studies. The development of several complex analytical approaches in impact evaluation, in addition to other contemporary developments and new challenges in various disciplines, is opening up new opportunities for exploring large databases originally collected for different purposes and is expanding its use for conducting research.

In 2001, the Brazilian government created the unified registry for social programmes ‘Cadastro Único’. The registry was greatly expanded and, by 2003, it became the main registry for the implementation and management of new and existing social programmes.^11^ As of 2018, the electronic database of the Cadastro Único comprised individual records of over 130 million people who had applied at any time for social benefits, of whom 74 million individuals were active in the database in that year.^12^ The extensive coverage of this social registry, the availability of individual data and the possibility of linkage to other health care data made it possible to design a cohort to evaluate the impact of the BFP and other social protection programmes on health and other outcomes. The creation of the 100 Million Brazilian Cohort by the Center for Data and Knowledge Integration for Health (CIDACS) was motivated by the availability of high-quality but dispersed social and health databases in Brazil and the need to integrate data and evaluate the impact of policies aiming to improve the social determinants of health (e.g. social protection policies) on health outcomes, overall and in subgroups in a dynamic Brazilian cohort in a middle-income country with high inequalities.

This study was done under the Declaration of Helsinki and the Brazilian research regulation agency and was approved by the Ethics Committee of Instituto Gonçalo Muniz from Oswaldo Cruz Foundation (Fiocruz) (1.612.302).

## Who is in the cohort?

The National Unified Register for Social Programmes (Cadastro Único para Pro- gramas Sociais or Cadastro Único) identifies low-income families who have applied for social assistance in Brazil. Since 2003, Cadastro Único has become the main instrument used by the Brazilian government to assess the family's eligibility for social programmes. To be enrolled in Cadastro Único, families have to have an income of up to half minimum wage per capita (approximately USD125 in 2020) or a total family income of three minimum wages (approximately USD750). Demographic and socioeconomic information on all family members is provided by a designated representative of the family, who must be at least 16 years old and should preferably be a woman. The baseline of the 100 Million Brazilian Cohort comprises individuals registered in Cadastro Único for the first time. It includes individuals who have applied to receive any social benefit since 2001, although not all individuals have necessarily been successful in this application. Information on receipt of the largest social benefit in Brazil, the Bolsa Familia programme, is only available from 2004 onwards.^11^ It is important to note that there was is a permanent effort to actively enrol extreme poor and poor families in Cadastro Único.^12^ Applicants answer a detailed form that collects demographic, economic and social information of each member of the family and on family and household characteristics.

**Figure 1:**
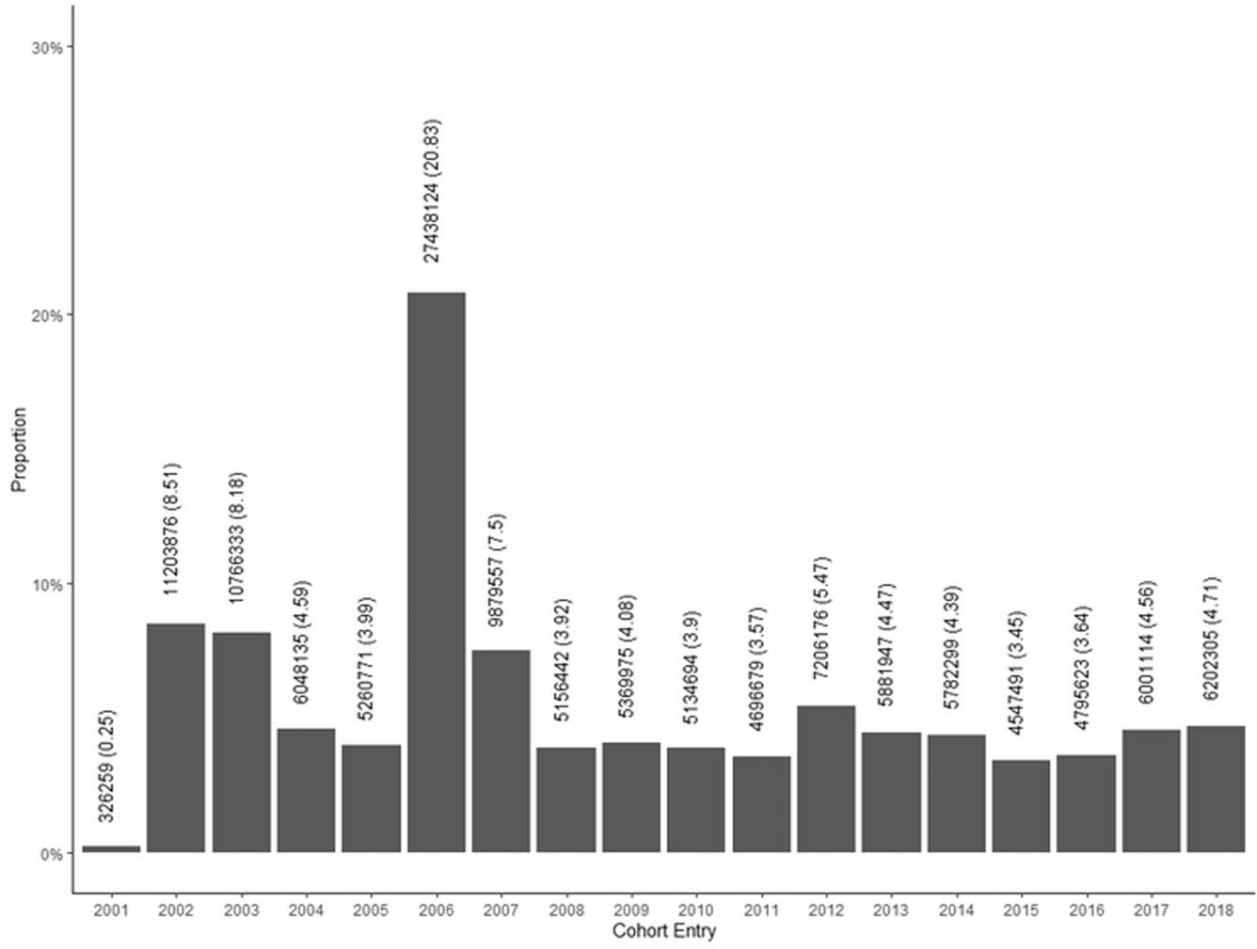
Number of individuals in the 100 Million Brazilian Cohort by entry calendar year

The baseline dataset includes a total of 131 697 800 individuals, about 62% of the Brazilian population, who entered at different periods from 2001 to 2018 (Figure 1). Once enrolled in the Cohort, individuals will remain in the Cohort even if they improve their socioeconomic conditions and are not longer eligible for social support. Nevertheless, individuals will be followed up to 31 December 2018, until they die (i.e. by linking individuals with death registry) or until they present an specific outcome of interest to be defined using linked data. There is an ongoing arrangement with the Ministry of Citizenship to get this baseline regularly updated, as the register at Cadastro Único is continuous.

The Cohort comprises approximately 11 million individuals enrolled in 2001 and 2002 for social benefits (i.e. prior to the official launch of Cadastro Único in 2003); 22 million people registered in the first 3 years (2003–05); 27.4 million people registered in 2006; over 9 million individuals enrolled in 2007; and an average of 5 to 6 million new applicants per year enrolled from 2008 to 2018.

**Figure 2:**
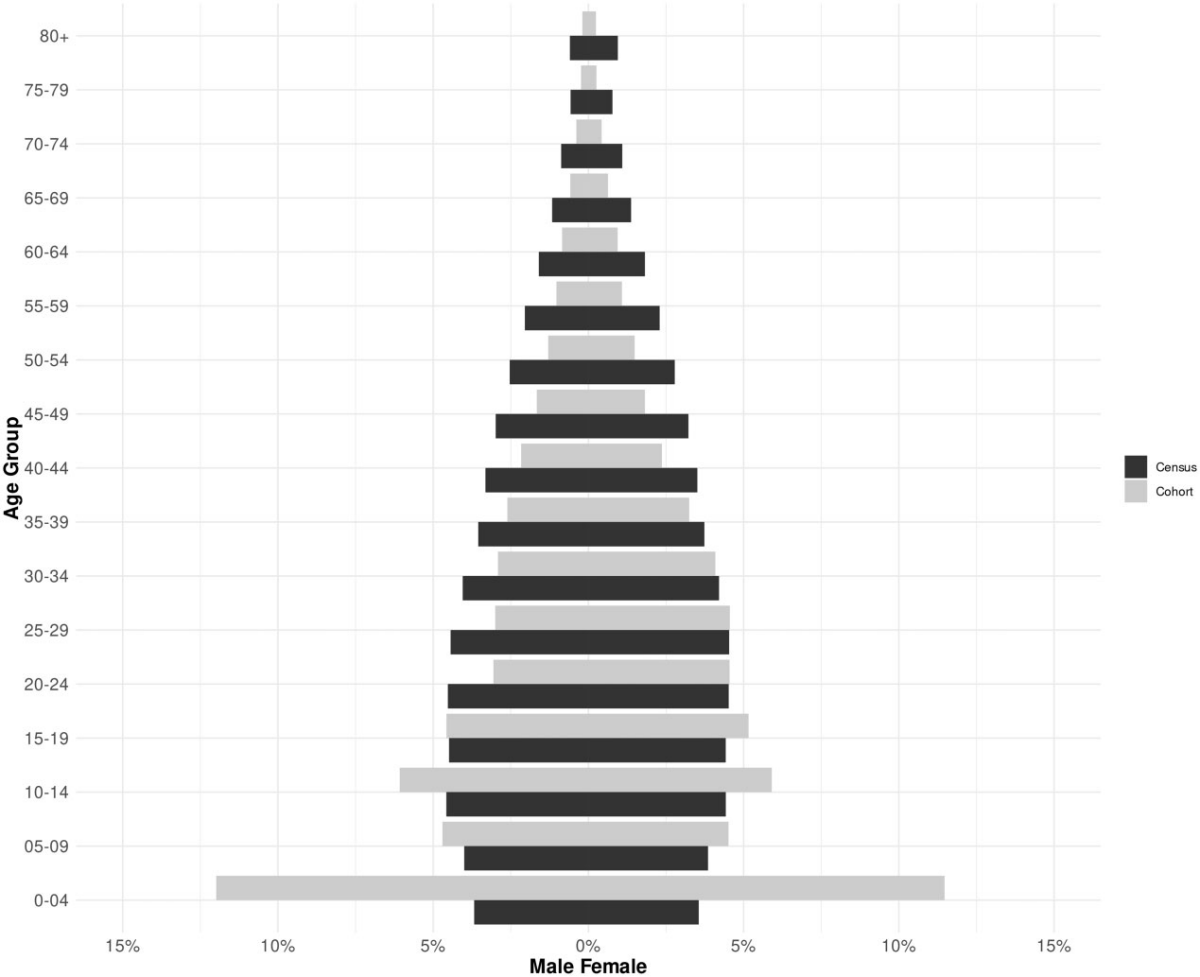
Comparison of the age and sex structure of the 100 Million Brazilian Cohort population (*N* = 131 697 800) and the Brazilian population for 2010 (*N* = 190 732 694)

When comparing the sex and age distribution of the 100 Million Brazilian Cohort at baseline (i.e. at the moment individuals were first enrolled—any time over 2011–2018) with that of Brazilian 2010 Census population, the Cohort population is over-representative of children and young people and under-representative of older adults and males (Figure 2). This is mostly the case because a large proportion of the Cohort are enrolled with the objective to apply to the Bolsa Familia Programme which targets pregnant/breastfeeding woman and children. Most of the population that were ever enrolled in the 100 Million Brazilian Cohort identified themselves for race/ethnicity as Brown (55.8%), 30.7% were self-identified as White, 6.6% as Black, 0.4% as from Asian ancestry and 0.6% as Indigenous (Table 1). By analysing children aged 6 to 15 years of age at baseline, it was found that 86.7% were attending school (84.1% attending government school), 2.4 % were not attending (but had already attended) and 3.4% had never attended. Similarly, 52.2% of the individuals aged 16 years or older had completed elementary or middle school, 19.2% had completed high school and 12.2% had never attended school (Table 1).

**Table 1:** Social and demographic characteristics of individuals ever enrolled in the 100 Million Brazilian Cohort (*N* = 131 697 800)

Characteristics	*N*	%
Sex (*N *= 131 697 800)		
Male	62 671 718	47.6
Femal	69 026 082	52.4
Race/ethnicity (*N* = 131 697 800)		
White	40 473 922	30.7
Black	8 696 072	6.6
Brown	73 478 974	55.8
Asian	537 530	0.4
Indigenous	769 372	0.6
Missing	7 741 930	5.9
Residence (*N* = 131 697 800)		
Urban	96 864 452	73.5
Rural	31 425 827	23.9
Missing	3 407 521	2.6
Attends/attended school for children 6–15 years of age (*N* = 25 188 678)		
Yes, state school	21 186 306	84.1
Yes, private school	655 306	2.6
No, but have already attended	592 555	2.4
No, have never attended	856 785	3.4
Other	131 161	0.5
Missing	74 569	0.3
Schooling for individuals >16 years of age (*N *= 70 063 532)		
Never attended	8 577 155	12.2
Preschool	120 598	0.2
Literacy year (year before elementary school)	580 315	0.8
Elementary school	20 635 490	29.5
Middle school	15 924 112	22.7
High school	13 456 622	19.2
Higher education	1 356 688	1.9
Missing/invalid	9 412 552	13.4
Disability (*N* = 131 697 800)		
Blindness	246 606	0.2
Deafness	117 116	0.1
Physical	1 357 347	1.0
Mental	976 576	0.7
No disabilities	129 000 155	98.0
Type of domicile (*N* = 131 697 800)		
Private permanent	120 814 360	91.7
Private improvised	1 897 241	1.5
Collective	306 097	0.2
Other	2 505 140	1.9
Missing	6 174 962	4.7
Building material of the house (*N* = 131 697 800)		
Brick	96 076 069	73.0
Coated mud	3 436 218	2.6
Uncoated mud	3 324 122	2.5
Wood	14 669 500	11.1
Other	8 342 286	6.3
Missing	5 849 605	4.5
Water supply (*N* = 131 697 800)		
Public	89 395 624	67.9
Wells/fountain	26 822 660	20.4
Other	9 630 292	7.3
Missing	5 849 224	4.4
Sewage system (*N* = 131 697 800)		
Public	55 862 496	42.3
Septic tank	18 994 811	14.4
Rudimentary tank	32 003 524	24.3
Ditch	14 663 738	11.1
Other	1 959 900	1.5
Missing	8 213 331	6.2
Energy (*N* = 131 697 800)		
Metered	100 516 834	76.3
Community metered	7 044 200	5.4
Unmetered	7 531 692	5.7
Gas lamp	2 972 535	2.3
Candle	2 334 425	1.8
Other	5 449 794	4.1
Missing	5 848 320	4.4
Garbage (*N* = 131 697 800)		
Public system	94 010 759	71.4
Burned/buried	22 596 523	17.2
Open ditches	7 856 565	6.0
Other	1 384 283	1.0
Missing	5 849 670	4.4

At baseline, the majority of the population was living in urban area (73.5%), in private permanent housing (*n* = 120 814 360, 91.7%) and in buildings that were predominantly made of bricks (73.0%) (Table 1). Furthermore, 67.9% of the baseline population were served by the public water supply, 42.3% by the public sewage and 71.4% by the public waste collection system.

## How often have they been followed up?

**Figure 3:**
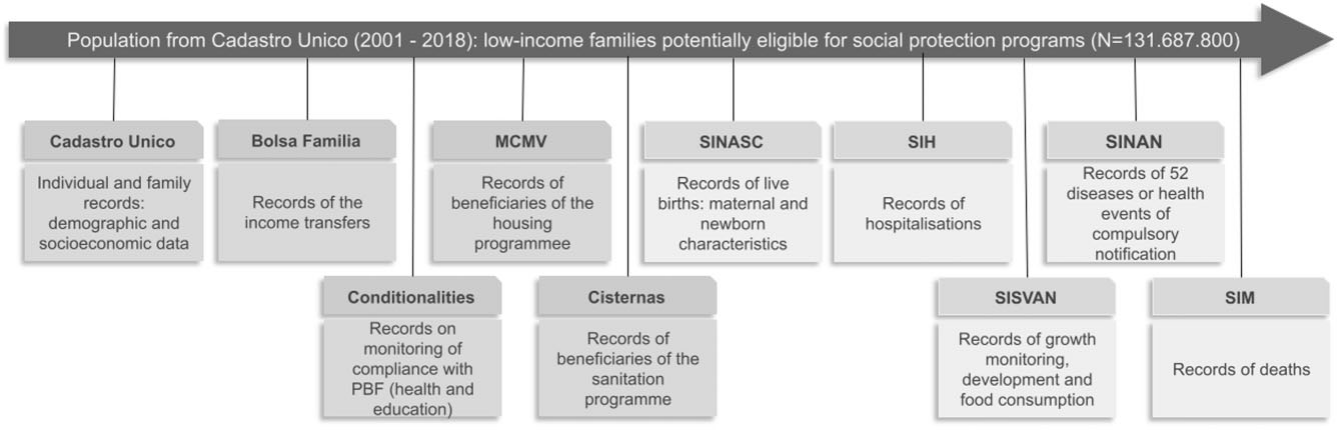
Main data sources linked to build the 100 Million Brazilian Cohort. MCMV—Minha Casa Minha Vida housing programme, SINASC—Sistema de Informação sobre Nascidos Vivos (Live Birth Information System), SIH—Sistema de Informações Hospitalares (Hospital Information System), SISVAN—Sistema de Vigilância Nutricional (Food and Nutrition Surveillance System), SINAN—Sistema de Informação de Agravos de Notificação (Information System for Notifiable Diseases), SIM—Sistema de Informação sobre Mortalidade (Mortality Information System)

Individuals’ follow-up is determined by the baseline registry up to the assessment of health outcomes obtained through linkage with health databases which has been continued by transfer to CIDACS upon agreements with the Brazilian Government (Table 2, Figure 3). At the first registration in Cadastro Único, each individual is allocated a unique Social Information Number (NIS). Social protection programmes databases (e.g. BFP, Minha Casa Minha Vida) also contain the NIS number for each beneficiary. Hence, individual records from Cadastro Único and social protection programmes databases can be linked deterministically using exact matching. Individuals registered in Cadastro Único also have a household identifier; hence, once the family member responsible for receiving BFP benefit in the baseline data is identified, it is also possible to assign the benefit to all members of a household. This linkage is performed for each year of BFP benefit, to take into account changes in the household structure. According to Cadastro Único regulations, each participant is required to update their socioeconomic information every 2 years as long as they are a candidate for receiving one of the government's social protection programmes. The registry update is mandatory for those who are receiving a benefit and for those who had their benefits turned down but want to reapply.

**Table 2:** Structure and main components of the 100 Million Cohort, sources of data and relevant variables to be linked

Components	Data source	Period	Relevant variables
Baseline	Cadastro Único (CadÚnico)	2001–18	Demographic and socioeconomic characteristics, family composition, entry date
Intervention (exposure)	Cash Transfer Programme-Bolsa Familia programme (BFP) payments	2004–18	Start and end of data receipt of benefit, total value by family, age, and number of months received
	BFP Conditionalities on health	2008–18	Follow-up situation, frequency of the health services
	BFP Conditionalities on education	2008–18	Follow-up situation, frequency in school
	Housing Programme (MCMV)^a^	2009–17	Marital status, date of receipt, monthly family income, municipality, date of birth, name of the enterprise and subprogramme
	Cisternas	2011–16	Municipality of residence, state, semi-arid zone
Outcomes	Death records (SIM)^b^	2000–18	Type of death, date of death, date of birth, sex, race, education, duration of the pregnancy, single or multiple pregnancy, type of delivery, age of mother, gestational age, birthweight, cause of death
	Birth records (SINASC)^c^	2001–18	Mother's data of birth, education, marital status, number of children and abortions, number of prenatal consultations, date of latest menstruation, duration of gestation, type of pregnancy, type of delivery and child's date of birth, sex, race, birthweight, presence of anomaly, who performed the childbirth among others
	Notifiable disease registry (SINAN)^d^	2001–18	Information on compulsory notifiable diseases, for example, tuberculosis, dengue, leprosy and syphilis
	Hospital admissions (SIH)^e^	2000–18	Municipality of residence, medical specialty, hospital unit, type of care, race/colour, reason for hospitalization, initial diagnosis, primary and secondary diagnosis (ICD), external causes
	Growth monitoring (SISVAN)^f^	200818	Nutritional status and anthropocentric indices, age, municipality of residence

a MCMV—Minha Casa Minha Vida housing programme.

b SIM—Sistema de Informação sobre Mortalidade (Mortality Information System).

c SINASC—Sistema de Informação sobre Nascidos Vivos (Live Birth Information System).

d SINAN—Sistema de Informação de Agravos de Notificação (Information System for Notifiable Diseases).

e SIH—Sistema de Informações Hospitalares (Hospital Information System).

f SISVAN—Sistema de Vigilância Nutricional (Food and Nutrition Surveillance System).

A non-deterministic linkage is performed using CIDACS-RL^13^ to link administrative health care datasets, such as mortality (SIM), disease notification (SINAN), birth information (SINASC) and nutrition status and measurements (SISVAN). CIDACS-RL is a record linkage tool designed to link large administrative data from Brazil using an individual's name, maternal name, gender, municipality of residency and date of birth or age records.^13^ It enables the integration of outcomes (mortality, hospitalization, diseases and child and maternal outcomes) into the Cohort baseline.^14–17^

## What has been measured?

Individuals registered with the baseline of the 100 Million Brazilian Cohort have a range of socioeconomic and demographic characteristics measured at individual and family levels (Table 1), which serve as exposures for studying the socioeconomic determinants of health. Also, the fact that families share an identifier allows study of siblings, intrahousehold transmission and intergenerational effects of poverty. In addition, exposures to several social interventions are measured through the linkage of the Cohort baseline with social protection programmes databases. So far, the Cohort baseline has been linked with BFP (receipt and conditionalities) and Minha Casa Minha Vida programmes receipt, but will be further linked with Cisternas receipt and have the potential to be linked with many others (Table 2, Figure 3).

The BFP, implemented in 2004, originally set the eligibility criteria at a fixed monthly per capita household income of 100 Brazilian reais (BRL) (USD25) for poor families and BRL50 (USD12) for extremely poor families. Since then, there have been several updates in the programme thresholds to account for inflation and increases in the living costs. BFP covers 23% of the Brazilian population with the benefits ranging from BRL41 (USD10) to a maximum of BRL300 (USD75) per month. The mother, when present, must receive the monthly payment on behalf of the whole family.^18^ The BFP database, when linked with the Cohort baseline, provides information on which low-income families received BFP benefits, the starting and end date of the receipt and the values that each family received over time. Several studies have reported that this programme is associated with improved socioeconomic determinants of health and better health-related outcomes, and have found that municipalities with higher coverage of BFP are associated with lower poverty and reduced inequalities,^19^ lower crime rates,^20^^,^^21^ lower suicide rates,^22^ lower leprosy^23^ and tuberculosis incidence,^24^ and lower child mortality and hospital admissions.^25^

BFP receipt is subject to families’ compliance with conditionalities. Conditionalities are commitments made by beneficiary families with the aim of increasing the access by the poorest Brazilians to the social rights of health, education and social assistance, which can further increase the chances of breaking out of the intergenerational poverty cycle. The conditionalities include: (i) enrolment and minimum monthly school attendance of 85% for children aged 6 to 15 years and 75% for adolescents aged 16 and 17 years; (ii) children up to 7 years of age must complete vaccination and growth monitoring; and (iii) beneficiary families with pregnant women and breastfeeding mothers should follow a health and nutrition agenda (pre- and postnatal care, vaccination and health and nutrition surveillance).^26^ The BFP conditionality database provides the information on educational enrolment or attendance, vaccination history, growth and nutrition monitoring of children and young adults and prenatal and postnatal care of pregnant and lactating mothers.

The assessments of health outcomes over time compose the follow-up measurements of the 100 Million Brazilian Cohort (Figure 3). To evaluate individual health outcomes, the Cohort baseline is linked to national health registries (e.g. birth or death registries, disease notification, hospitalisations, and food and nutrition data including assessment of anthropometric measures and food consumption markers). Currently, the Cohort baseline and other relevant databases are being geocoded, allowing a more granular spatial analysis below the municipality level and to contribute to a better understanding of the inequities and material deprivations that act on the poorest populations.

The content of the Cohort to be analysed will vary according to each research question and the outcome investigated. The possible inclusion of new interventions or outcomes will increase the analytical possibilities by allowing investigation of not only the isolated impact of different interventions but also the joint effect of two or more interventions.

## What has it found?

Some studies have already used the 100 Million Brazilian Cohort to investigate the social determinants of health and to evaluate the impact of social protection on similar outcomes (Table 3), and many are still being conducted. By linking the Cohort baseline with BFP and leprosy registries, some studies have stressed that poor socioeconomic characteristics are important determinants of individuals having leprosy^27^ and leprosy-related disabilities^28^ and are associated with leprosy treatment default.^29^ In addition, it was suggested that BFP is associated with lower leprosy incidence in high-burden municipalities in the country^30^ and that leprosy patients receiving BFP have higher likelihood of adhering to leprosy treatment and cure.^31^ More recently, by linking the Cohort baseline with birth records, a study has also investigated the socioeconomic determinants of low birthweight^32^ and small or large size for gestational age.^33^ Several other studies are in progress involving the social determinants or impact of social protection on child and maternal health, suicide, homicides, leprosy, tuberculosis, HIV, cancer and cardiovascular diseases.^34^^,^^35^ The ongoing linkage of hospitalization data will enhance the previous analyses and open possibilities for multi-morbidity studies. In addition, the coverage of long periods comprising before and after major economic changes and crisis in the past decade, allows study of the impact of those changes on health and on the economic fragility (e.g. access to Bolsa Família, income, school attendance). In the future, the Cohort will also enable evaluation of the impact of current government policies and changes made, as data become available for linkage.

**Table 3:** Publications using the 100 Million Brazilian Cohort

Author/year	Title	Journal	Exposure	Results	Doi
Ramos DO *et al.* 2021	Conditional cash transfer program and child mortality, a cross-sectional analysis nested within the 100 Million Brazilian Cohort	*Plos Medicine*	BFP receipt	Child mortality	10.1371/journal.pmed.1003509
Ferreira AJ *et al*. 2021	Evaluating the health effect of a Social Housing programme, Minha Casa Minha Vida, using the 100 million Brazilian Cohort: a natural experiment study protocol	*BMJ Open*	Minha Casa Minha Vida (MCMV) housing programme receipt	Leprosy incidence; tuberculosis incidence; cardiovascular, diabetes and all-cause mortality	10.1136/bmjopen-2020-041722
Pescarini JM *et al*. 2020	Evaluating the impact of the Bolsa Familia conditional cash transfer program on premature cardiovascular and all-cause mortality using the 100 million Brazilian cohort: a natural experiment study protocol	*BMJ Open*	BFP receipt	Cardiovascular, and all-cause mortality	10.1136/bmjopen-2020-039658
Falcão I *et al.* 2021	Factors associated with small- and large-for- gestational-age in socioeconomically vulnerable individuals in the 100 Million Brazilian Cohort	*American Journal of Clinical Nutrition*	Baseline characteristics	Small or large for gestational age/birth registration (SINASC)	10.1093/ajcn/nqab033
Sanchez MN *et al.* 2021	Physical disabilities caused by leprosy in 100 million cohort in Brazil	*BMC Infectious Diseases*	Baseline characteristics	Leprosy cases with disabilities/leprosy records (SINAN)	10.1186/s12879-021-05846-w
Falcão I *et al.* 2020	Factors associated with low birth weight at term: a population-based linkage study of the 100 million Brazilian cohort	*BMC Pregnancy Childbirth*	Baseline characteristics	Low birthweight/birth registration (SINASC)	10.1186/s12884-020-03226-x
Pescarini JM *et al*. 2020	Conditional Cash Transfer Programme and leprosy incidence: analysis of 12.9 million families from the 100 Million Brazilian Cohort	*American Journal of Epidemiology*	BFP receipt	New cases of leprosy/leprosy records (SINAN)	10.1093/aje/kwaa127
Teixeira CSS *et al*. 2020	Incidence of and factors associated with leprosy among household contacts of patients with leprosy in Brazil	*JAMA Dermatology*	Baseline characteristics	New cases of leprosy/leprosy records (SINAN)	10.1001/jamadermatol.2020.0653
Pescarini JM *et al*. 2020	Effect of a conditional cash transfer programme on leprosy treatment adherence and cure in patients from the nationwide 100 Million Brazilian Cohort: a quasi-experimental study	*Lancet Infectious Diseases*	BFP receipt	Leprosy treatment outcomes/leprosy records (SINAN)	10.1016/S1473-3099(19)30624-3
de Andrade KVF *et al*. 2019	Geographic and socioeconomic factors associated with leprosy treatment default: An analysis from the 100 Million Brazilian Cohort	*PLoS Neglected Tropical Diseases*	Baseline characteristics	Leprosy treatment outcomes/leprosy records (SINAN)	10.1371/journal.pntd.0007714 (Erratum in 10.1371/journal.pntd.0008723)
Nery JS *et al.* 2019	Socioeconomic determinants of leprosy new case detection in the 100 Million Brazilian Cohort: a population-based linkage study	*Lancet Global Health*	Baseline characteristics	New cases of leprosy/leprosy records (SINAN)	10.1016/S2214-109X(19)30260-8

SINASC—Sistema de Informação sobre Nascidos Vivos (Live Birth Information System); SISVAN—Sistema de Vigilância Nutricional (Food and Nutrition Surveillance System); SINAN—Sistema de Informação de Agravos de Notificação (Information System for Notifiable Diseases).

## What are the main strengths and weaknesses?

The 100 Million Brazilian Cohort has several strengths. The conceptualization and organization of the Cohort is innovative both in Brazil and worldwide, as it links health and social data coming from various government sectors. Also, the Cohort is a unique resource contributing to the study of the social determinants of health and the effects of social protection policies focused on these determinants on specific health outcomes. The quality and the longitudinal organization of the data are advantageous for making robust inferences. Individuals follow-up could start from 2001 onward, and as most social policies started in 2003, there is a long period of follow-up that will continue prospectively; hence long-term effects could also be studied in the future. It also enables the addition of new exposures or outcomes and the study of outcomes at different times from the exposure. Second, the large size of the Cohort will also allow the exploration of effects on less common health outcomes and their variation in sub-population subgroups, including vulnerable groups (e.g. ethnic minorities or individuals living in poorer or rural areas, and isolated populations such as Indigenous people and communities originally organized by fugitive slaves of African descent). The Cohort will, for example, allow us to explore intersectional risk factors of health outcomes and detailed interactions (e.g. fine age strata, gender and racial interactions, and the effect of combined social policies). Third, the use of administrative data eliminates the risk of recall bias, which is a problem if data collection relies on self-reports of service use (e.g. hospitalization or birth). Fourth, the linkage has been conducted with a robust and accurate software developed in house (CIDACS-RL), and a specialized team evaluates each linkage performed at CIDACS.^13^^,^^15–17^

Although promising, there are some limitations that must be considered when analysing and interpreting the 100 Million Brazilian Cohort. The Cohort has as its baseline the Cadastro Único, an administrative database not designed for research purposes. Hence, we have a considerable proportion of missing values in variables that are not mandatory in Cadastro Único, such as education, occupation of the family members and some characteristics of the household. Nevertheless, the description of all individuals in the household (e.g. sex, age and ethnicity) and variables such as income, key variables that are used as eligibility criteria for social programmes have good completeness. Although the characteristics of people enrolled at Cadastro Único represent individuals seeking social benefits, which limits the generalizability of results to the entire population of Brazil, they are a very solid and unbiased representation of the poorer half of the population. Finally, the linkage of large databases to generate the 100 Million Brazilian Cohort poses several challenges, such as computational complexity, the limited numbers of identifiers and the absence of a unique identifier for both social and health databases. As the linkage process can introduce measurement bias of the outcomes, which is a general characteristic of administrative data cohorts, that requires continuous quality and accuracy checks.

## Can I get hold of the data? Where can I find out more?

Currently, only national and international researchers who collaborate with CIDACS and authorized staff from government agencies can access de-identified or anonymized linked data. These individuals and organizations must be committed to advancing scientific knowledge or generating evidence for public policy formulation. We particularly encourage PhD students and early-career researchers to apply to use the data. Researchers can access data relevant to their proposed study objectives exclusively via an authorized virtual private network (VPN) with two-factor authentication (mobile token) and the data are stored, processed and managed in the high-level security system that provides a custom virtual machine (i.e., access to a safe heaven with adequate storage and processing for handling the dataset provided).

Any person who wishes to receive authorization must: (i) be affiliated to CIDACS or be accepted as collaborators; (ii) present a detailed research project together with approval by an appropriate Brazilian institutional research ethical committee; (iii) provide a clear data plan restricted to the objectives of the proposed study and a summary of the analyses plan intended to guide the linkage and or data extraction of the relevant set of records and variables; (iv) sign terms of responsibility regarding the access and use of data; and (v) perform the analyses of datasets provided using the CIDACS data environment, a safe and secure infrastructure that provides remote access to de-identified or anonymized datasets and analysis tools. For more information, please visit the CIDACS website [https://cidacs.bahia.fiocruz.br/] or contact us via email [cidacs@fiocruz.br].

## Funding

CIDACS received core support from Health Surveillance Secretariat, Ministry of Health, Brazil; Fundação de Apoio a Pesquisa do Estado da Bahia (FAPESB); Wellcome Trust (grant number 202912/Z/16/Z); Financiadora de Estudos e Projetos- FINEP, Secretaria de Ciência e Tecnologia do Estado da Bahia-SECTI, Grand Challenges Brazil grant, Ministerio de Ciencia, Tecnologia e Inovação - MCTI / Conselho Nacional de Desenvolvimento Científico e Tecnológico -CNPq / Ministério da Saude (Brazil) -MS / Secretaria de Ciência, Tecnologia, Inovação e Insumos Estratégicos em Saúde -SCTIE / Departamento de Ciência e Tecnologia - DECIT / Bill and Melinda Gates Foundation (call No. 47/2014). EPS is funded in part by the Wellcome Trust [grant number 213589/Z/18/Z]. This work was supported by the British Council Newton Fund (527418645).

## Author contributions

All authors have contributed to the concept and design of the study; M.Y.I., G.C.G.B., D.A., S.S. and D.R. contributed to implementing the cohort. M.L.B., L.C.R. and M.S.A. wrote the first draft of the manuscript with contribution from M.Y.I., R.F., G.C.G.B., B.A.A. and M.E.B. to sections of the manuscript. E.S.P. and J.P. updated the manuscript data. All authors have contributed to manuscript revision, have read and have approved the submitted version.

## Conflict of interest

None declared.
